# Hematologic and Renal Trends in Cats Undergoing Continuous Renal Replacement Therapy: A Retrospective Case Series with Molidustat Use

**DOI:** 10.3390/vetsci13090973

**Published:** 2026-09-16

**Authors:** Gippeum Lee, Jeongmin Lee, Kun-Ho Song

**Affiliations:** 1 Department of Veterinary Internal Medicine, College of Veterinary Medicine, Chungnam National University, Daejeon 34134, Republic of Korea; vetmania@cnu.ac.kr (G.L.); memo7677@cnu.ac.kr (J.L.); 2 Korea Animal Medical Center, Cheongju 28651, Republic of Korea

**Keywords:** continuous renal replacement therapy, acute kidney injury, anemia, molidustat, hypoxia-inducible factor prolyl hydroxylase inhibitor, cat

## Abstract

Continuous renal replacement therapy (CRRT) can precipitate anemia in critically ill patients, but temporal hematologic trends and anemia management strategies in cats remain poorly characterized. Medical records of three client-owned cats treated with CRRT for severe acute kidney injury with evidence of pre-existing chronic kidney disease at a single referral center were retrospectively reviewed. Hematologic variables (hematocrit, hemoglobin concentration, absolute reticulocyte count) and renal parameters (blood urea nitrogen, creatinine, phosphorus) were evaluated before, during, and after CRRT, along with the timing of CRRT sessions, whole blood transfusions, erythropoiesis-stimulating agent administration, and adjunctive molidustat use. All three cats demonstrated a progressive decline in hematocrit during the CRRT period despite repeated whole blood transfusions, with only transient post-transfusion improvement. Blood urea nitrogen and creatinine concentrations decreased promptly following CRRT initiation but showed variable rebound patterns during follow-up. Absolute reticulocyte counts increased after CRRT discontinuation in two cats, indicating preserved or recovering erythropoietic activity, while one cat with concurrent inflammatory and infectious disease showed a minimal response. Progressive anemia occurred consistently despite transfusion support, highlighting the multifactorial nature of anemia during feline CRRT. These descriptive findings provide clinically relevant insight into anemia progression during feline CRRT and may inform the design of future prospective studies.

## Simple Summary

Kidney failure in cats can be treated with a machine-based blood-filtering therapy called continuous renal replacement therapy, but this treatment is often followed by low red blood cell counts (anemia). This report describes three cats with sudden worsening of long-standing kidney disease that were treated with this therapy at one veterinary hospital. Alongside standard care such as blood transfusions and a red-blood-cell-stimulating hormone, all three cats also received an oral medication called molidustat, which is intended to help the body produce more red blood cells. We tracked each cat’s blood counts and kidney values before, during, and after treatment. Kidney values improved soon after treatment began in all three cats, although some cats showed a temporary worsening between treatment sessions. Despite blood transfusions, all three cats became more anemic over the course of treatment, and only some showed signs of new red blood cell production afterward. Because several treatments were given together over a short period, we could not determine whether molidustat itself affected red blood cell recovery. These findings describe what happened in these cats and may help veterinarians plan future research into managing anemia in cats receiving this therapy.

## 1. Introduction

Acute kidney injury (AKI) in cats is associated with substantial morbidity and mortality [1,2], and timely diagnosis and aggressive management are critical determinants of outcome [3]. In clinical practice, AKI frequently occurs in cats with evidence of pre-existing chronic kidney disease [4,5,6], resulting in an unstable metabolic and hematologic environment. These patients are at increased risk of severe azotemia, uremic complications, and fluid and electrolyte derangements, which may necessitate advanced renal support [3].

In severely affected feline patients, particularly those with refractory uremia, fluid overload, or electrolyte imbalances, continuous renal replacement therapy (CRRT) has emerged as an important therapeutic option [4]. CRRT allows gradual solute and fluid removal and is often favored in hemodynamically unstable patients [3]. Although the use of CRRT in small animal medicine, including feline patients, has increased in recent years, published reports describing associated complications and longitudinal clinical outcomes in cats remain limited [3].

Anemia is a well-recognized complication of CRRT in both human and veterinary patients. The development of anemia during CRRT is multifactorial and has been attributed to repeated blood sampling, extracorporeal circuit-related blood loss, anticoagulation, inflammation, and disturbances in iron metabolism [7,8,9,10]. In cats, whose circulating blood volume is relatively small, these factors may impose a disproportionately greater hematologic burden during extracorporeal therapies [3]. Consequently, progressive anemia may develop despite transfusion support and standard anemia management strategies. Because the extracorporeal circuit volume used during CRRT may represent a substantial proportion of the total circulating blood volume in cats, hematologic responses during treatment may differ from those observed in larger species [3].

Management of anemia in critically ill patients undergoing CRRT typically relies on whole blood transfusions and, in some cases, erythropoiesis-stimulating agents (ESAs) [7,11]. While these interventions may transiently improve oxygen-carrying capacity, they are often insufficient to prevent ongoing anemia during prolonged or repeated CRRT [7]. In the human critical care literature, anemia frequently persists or worsens in patients receiving CRRT despite transfusion and ESA therapy [7], underscoring the complex and multifactorial nature of anemia in this setting.

Recently, hypoxia-inducible factor prolyl hydroxylase inhibitors (HIF-PHIs) have been introduced as a novel therapeutic class for the management of anemia associated with chronic kidney disease [12]. Molidustat stimulates a dose-dependent erythropoietic response in healthy cats [13] and may be an alternative for the management of CKD-associated anemia in cats [14]. However, data regarding hematologic responses in cats undergoing CRRT for acute kidney injury, particularly in the presence of severe systemic illness and repeated extracorporeal interventions, are extremely limited [15].

CRRT represents a highly dynamic treatment environment characterized by continuous extracorporeal circulation, repeated interventions, and profound metabolic and inflammatory fluctuations [3,16]. Under these conditions, hematologic trends may differ substantially from those observed in stable CKD patients, and interpretation of anemia management responses is inherently challenging. Descriptive reporting of hematologic and renal trends in feline patients undergoing CRRT is therefore of clinical relevance.

The objective of this retrospective observational study was to describe temporal changes in hematologic and renal parameters in cats undergoing continuous renal replacement therapy for acute kidney injury with evidence of pre-existing chronic kidney disease, and to report clinical observations related to anemia management during this high-risk treatment period.

## 2. Materials and Methods

### 2.1. Study Design

This study was designed as a retrospective observational study. Medical records of client-owned cats treated with continuous renal replacement therapy (CRRT) at a single referral center were reviewed to evaluate temporal hematologic and renal trends during and after extracorporeal renal support.

### 2.2. Case Selection

This retrospective descriptive case series is based on three clinical cases in which continuous renal replacement therapy (CRRT) was performed for severe acute kidney injury with evidence of pre-existing chronic kidney disease, and in which molidustat was used as part of clinical anemia management during the treatment course. These cases were subsequently reviewed retrospectively to describe their serial hematologic and renal changes before, during, and after CRRT. Cats were not identified through systematic screening of a predefined cohort of CRRT-treated patients over a specified study period, and no formal inclusion or exclusion process with a defined denominator of screened cases was applied. Baseline characteristics and treatment overview for the three cats are summarized in Table 1.

### 2.3. CRRT Protocol

Continuous renal replacement therapy was performed using a double-lumen 8-Fr hemodialysis catheter placed in the right jugular vein for vascular access. Treatments were conducted using a Fresenius Multifiltrate System (Fresenius Medical Care AG & Co. KGaA, Bad Homburg, Germany) in pediatric continuous venovenous hemodialysis (CVVHD) mode with polysulfone dialyzers (Ultraflux AV paed, Fresenius Medical Care AG & Co. KGaA, Bad Homburg, Germany).

Because of the relatively small circulating blood volume of feline patients [17], the extracorporeal circuit and dialyzer, with an approximate priming volume of 60 mL, were primed using crossmatch-compatible whole blood prior to treatment initiation.

All systemic anticoagulant medications were discontinued during treatment, and anticoagulation of the extracorporeal circuit was achieved using a continuous-rate infusion of unfractionated heparin administered at 2.0–4.8 IU/h according to clinical requirements. Blood and dialysate flow rates were adjusted according to individual patient tolerance and clinical response throughout treatment sessions.

Treatment protocols were standardized across all cases. Case 1 underwent a single 6-h session using intermittent hemodialysis (IHD). Case 2 underwent three sessions with durations of 5, 8, and 6 h, respectively, consisting of one IHD session followed by two prolonged intermittent renal replacement therapy (PIRRT) sessions. Case 3 underwent three sessions with durations of 10, 8, and 6 h, all performed using PIRRT. No episodes of filter clotting or clinically apparent extracorporeal circuit blood loss were observed during any treatment session. Based on the body weight of the included cats (3.5–3.8 kg), the extracorporeal circuit volume represented approximately 23–31% of the estimated circulating blood volume.

### 2.4. Data Collection

Clinical, hematologic, and biochemical data were extracted from electronic medical records. Biochemical analyses, including BUN, creatinine, and phosphorus, were performed using a FUJI DRI-CHEM chemistry analyzer (Fujifilm Corporation, Tokyo, Japan). Complete blood counts, including HCT, hemoglobin, and absolute reticulocyte count, were performed using a Mindray BC-60 hematology analyzer (Shenzhen Mindray Animal Medical Technology Co., Ltd., Shenzhen, China). Point-of-care blood gas and electrolyte measurements, including the serial HCT values obtained during active CRRT sessions, were performed using a GEM Premier 5000 blood gas analyzer (Werfen, Bedford, MA, USA). Hematologic variables included hematocrit (HCT), hemoglobin concentration, and absolute reticulocyte count. Renal variables included blood urea nitrogen (BUN), serum creatinine, and serum phosphorus concentrations.

Treatment-related variables recorded included the timing and number of CRRT sessions, timing and volume of whole blood transfusions, use of erythropoiesis-stimulating agents, and adjunctive administration of molidustat as part of clinical anemia management. All medications and interventions were administered at the discretion of the attending clinician as part of routine patient care.

Day 0 was defined as the day of initiation of the first CRRT session for each cat. Because this was a retrospective clinical case series rather than a prospectively standardized study, the timing and frequency of laboratory measurements were determined by the clinical condition and treatment requirements of each patient and were therefore not identical among the three cats; multiple measurements were obtained on some CRRT treatment days, whereas testing frequency decreased as patients stabilized. AKI severity was considered within the International Renal Interest Society (IRIS) framework; because the urine output data required for formal IRIS AKI grading were not consistently documented, AKI severity is instead described using the serial BUN and creatinine values at the start and end of the CRRT course for each cat, as presented in Table 2. Based on serum creatinine concentration at presentation, all three cats met the IRIS threshold for CKD Stage 4 (the most severe of the four IRIS CKD stages; creatinine > 5.0 mg/dL). IRIS guidance specifies that CKD staging should be performed only in stable, well-hydrated patients once acute kidney injury has been ruled out or resolved; because these creatinine values were measured during an episode of acute-on-chronic kidney injury rather than in a stable state, this Stage 4 classification is presented descriptively to characterize the severity of azotemia at presentation, rather than as a formal chronic-phase IRIS substaging. Laboratory values were organized chronologically relative to Day 0 and the subsequent treatment course to facilitate evaluation of temporal trends. No interventions were performed solely for the purpose of data collection or analysis.

### 2.5. Outcome Assessment

The primary outcomes of interest were temporal changes in hematologic and renal parameters during and after CRRT. Analyses focused on descriptive patterns of anemia progression, reticulocyte response, and renal parameter fluctuations rather than absolute numerical comparisons or inferential statistical testing.

### 2.6. Data Presentation and Analysis

Data were summarized descriptively and visualized using line graphs to illustrate individual patient trajectories over time. Given the small sample size and observational nature of the study, no inferential statistical analyses were performed.

Detailed individual clinical data, including CRRT indications, treatment timelines, transfusion history, erythropoietic support, and serial laboratory values, are provided in the Supplementary Material to support interpretation of the reported trends.

During the preparation of this manuscript, the authors used Claude (Sonnet 5; Anthropic) to assist with language editing, organization and verification of the reference list, and consistency checking of reported statistical and numerical data. The authors reviewed and edited all AI-assisted output and take full responsibility for the content of the manuscript.

## 3. Results

### 3.1. Clinical Course

Case 1

An 8-year-old neutered female domestic shorthair cat presented with acute kidney injury and evidence of pre-existing chronic kidney disease. Due to rapidly worsening azotemia and persistent uremic signs refractory to medical management, a single session of continuous renal replacement therapy (CRRT) was performed, accompanied by a type A whole blood transfusion (40 mL).

Following CRRT, progressive anemia was observed, and on Day 2 (February 20) a single dose of darbepoetin alfa (1 µg/kg SC) was administered together with initiation of oral molidustat (2.5 mg/kg PO SID) as part of clinical anemia management. Hematocrit declined during hospitalization, while an increase in absolute reticulocyte count was observed after discontinuation of CRRT. Blood urea nitrogen (BUN) and serum creatinine concentrations decreased markedly following CRRT and subsequently showed partial stabilization during follow-up.

Case 2

A 12-year-old neutered male Persian cat with acute-on-chronic kidney disease and partial ureteral obstruction was transferred to the referral center specifically for continuous renal replacement therapy because of severe azotemia and uremia refractory to medical management. CRRT was initiated on the day of admission (14 March, Day 0), and a total of three CRRT sessions were performed during hospitalization (14 March, 16 March [Day 2], and 19 March [Day 5]). Each CRRT session was accompanied by a concurrent type A whole blood transfusion (40 mL).

Pre-CRRT laboratory values on Day 0 included HCT 31.7%, absolute reticulocyte count 5.5 K/µL, BUN 138.5 mg/dL, creatinine 7.4 mg/dL, and phosphorus 11.9 mg/dL. Progressive anemia developed despite transfusion support. Oral molidustat (2.5 mg/kg PO SID) was started on Day 0, administered as the first CRRT session was being initiated, and darbepoetin alfa (1 µg/kg SC) was administered on Day 1 (15 March) as an additional adjunctive therapy. Hematocrit declined throughout hospitalization, whereas reticulocyte counts increased following completion of CRRT. Blood urea nitrogen and serum creatinine concentrations decreased after each treatment session but demonstrated intermittent rebound patterns consistent with dialysis-dependent renal failure.

Case 3

A 10-year-old neutered male domestic shorthair cat (approximately 3.5 kg) of community/feral origin and with pre-existing chronic kidney disease presented with severe azotemia, hyperammonemia, and stuporous mentation. In the approximately two weeks preceding presentation, routine care by the cat’s regular caregiver had been disrupted, during which the cat developed severe anorexia, marked weight loss, and progressive dehydration. Initial laboratory findings included creatinine 6.97 mg/dL, BUN > 140 mg/dL (exceeding the analyzer’s upper measurement limit), symmetric dimethylarginine (SDMA) > 100 µg/dL, and phosphorus 7.31 mg/dL. The acute kidney injury was considered multifactorial, occurring in the setting of pre-existing CKD, prolonged anorexia, weight loss, and dehydration/hemodynamic compromise, together with severe concurrent hepatobiliary and pancreatic disease (cholecystitis, hepatic lipidosis, and pancreatitis); a single definitive cause was not established. Additional findings included a small volume of abdominal free fluid on ultrasonography, most notably around the hepatobiliary region and caudal to the spleen, whose specific etiology could not be determined in the setting of concurrent hepatobiliary, pancreatic, renal, and systemic disease; gastrointestinal dysmotility; and hypertrophic cardiomyopathy (HCM) stage B1.

Three PIRRT sessions were performed (10 h on 12 October [Day 0], 8 h on 14 October [Day 2], and 6 h on 18 October [Day 6]), each accompanied by a concurrent type A whole blood transfusion (40 mL, 40 mL, and 50 mL, respectively; total 130 mL). Molidustat (2.5 mg/kg PO SID) and darbepoetin alfa (1 µg/kg SC) were both administered on Day 0, concurrently with the first CRRT session and transfusion. Following the first CRRT session, BUN decreased from >140 mg/dL to 77.3 mg/dL and creatinine decreased from 6.97 to 2.81 mg/dL; renal parameters showed further improvement with intermittent rebound (BUN up to >140 mg/dL) over the subsequent sessions; and ammonia decreased from 330 to 89 µmol/L. Hyperammonemia had resolved by October 13 and stuporous mentation by 15 October.

Initial white blood cell (WBC) countwas markedly elevated (43.51 K/µL, with neutrophils 41.99 K/µL). HCT was 39.9% and absolute reticulocyte count was 102.2 K/µL at presentation; reticulocyte counts fell to 8.0–9.9 K/µL during the CRRT period before increasing to 44.7 K/µL after the third CRRT session, while HCT declined to approximately 20.8%. During the approximately one-month hospitalization, the cat subsequently developed nasal obstruction and conjunctival edema; PCR testing of conjunctival and nasopharyngeal swabs was positive for feline herpesvirus (FHV), feline calicivirus (FCV), and *Mycoplasma* spp., and the cat was treated with famciclovir and doxycycline. These findings reflected clinically apparent respiratory and ocular disease rather than confirmed systemic infection. The cat had a history of screening-test feline immunodeficiency virus (FIV) antigen positivity with a subsequent negative confirmatory PCR and a negative repeat screening test.

### 3.2. Hematologic and Renal Trends

**Figure 1:**
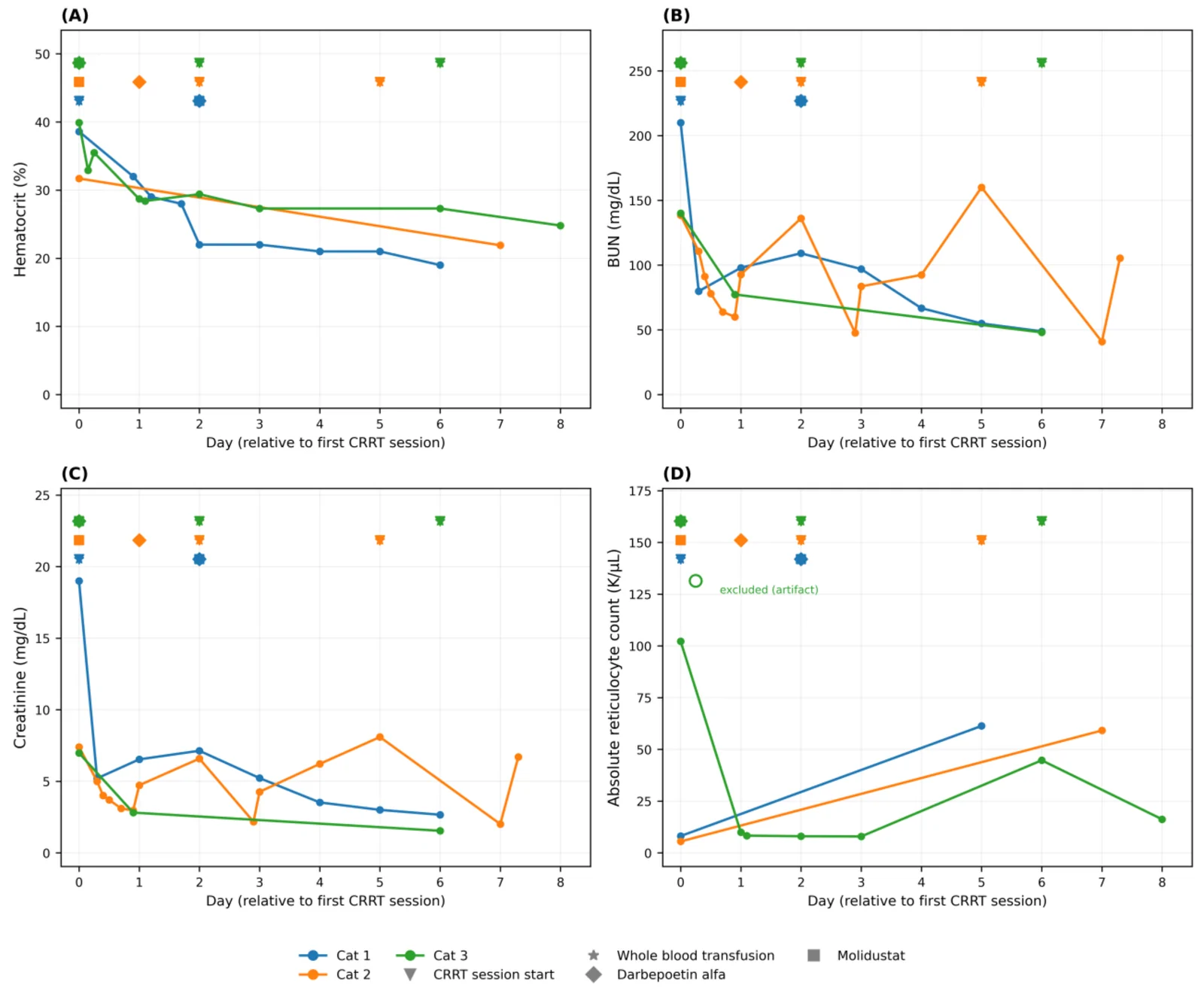
Hematologic and renal trends in three cats undergoing continuous renal replacement therapy (CRRT). Individual cats are distinguished by color: Cat 1 = blue, Cat 2 = orange, Cat 3 = green. Day 0 for each cat is defined as the day of the first CRRT session (Cat 1: 18 February 2025; Cat 2: 14 March 2025; Cat 3: 12 October 2024). Markers above each panel indicate the timing of the start of each CRRT session (inverted triangle), whole blood transfusion (star), darbepoetin alfa (diamond), and molidustat (square) administration, in the color corresponding to each cat. (**A**) Hematocrit (HCT) over time. (**B**) Blood urea nitrogen (BUN) concentrations over time. (**C**) Serum creatinine concentrations over time. (**D**) Absolute reticulocyte counts over time.

In all three cats, hematocrit (HCT) showed a consistent downward trend during the CRRT period despite repeated whole blood transfusions (Figure 1A). Cat 2 (orange) and Cat 3 (green), both of which underwent three CRRT sessions, exhibited stepwise decreases in HCT following each session. Cat 1 (blue), which received a single CRRT session, also demonstrated a post-CRRT decline in HCT. Although transient stabilization or mild increases in HCT were observed immediately after transfusion, these effects were not sustained, and overall anemia progression was observed in all cases.

Renal parameters responded promptly to extracorporeal therapy. Blood urea nitrogen (BUN) and serum creatinine concentrations decreased markedly immediately following CRRT initiation in all cats (Figure 1B,C). In Cats 2 and 3, subsequent rebound increases in BUN and creatinine were observed during follow-up, consistent with ongoing uremic production in the setting of dialysis-dependent acute kidney injury. In contrast, Cat 1 showed more sustained post-CRRT stabilization of azotemia, suggesting partial renal recovery.

Absolute reticulocyte counts demonstrated interindividual variability (Figure 1D). In Cats 1 and 2, reticulocyte counts increased following CRRT discontinuation, indicating regenerative erythropoietic responses despite preceding critical illness and transfusion exposure. Cat 2 showed the most pronounced reticulocyte increase among the three cats; this cat had received both darbepoetin alfa and molidustat, but because these treatments were administered concurrently with CRRT, transfusion, and recovery from critical illness, their independent contribution to this response cannot be determined. Cat 3 showed only a mild reticulocyte increase, which may reflect the influence of concurrent inflammatory and infectious disease, among other confounding factors present in this cat. Peak hematologic and renal parameters and overall trends for each cat are summarized in Table 2.

## 4. Discussion

This retrospective case series describes temporal changes in hematologic and renal parameters in three critically ill cats undergoing continuous renal replacement therapy (CRRT) for acute kidney injury, during which the hypoxia-inducible factor prolyl hydroxylase inhibitor (HIF-PHI) molidustat was used as part of multimodal anemia management. A consistent finding across all cases was the progression of anemia during CRRT despite transfusion support, while evidence of erythropoietic recovery was observed in two cats (Cat 1 and Cat 2) following discontinuation of CRRT.

CRRT is an essential life-support modality for managing severe AKI in both human and veterinary medicine; however, anemia is a well-recognized complication of this therapy [4]. The pathogenesis of CRRT-associated anemia is multifactorial—including repeated blood sampling, extracorporeal circuit-related blood loss, anticoagulation, inflammatory responses, and disturbances in iron metabolism—based primarily on evidence from human critical care and canine renal replacement therapy studies [16]. Comparable feline-specific mechanistic studies remain scarce, although cats are particularly vulnerable to anemia during intensive care [18]. In the present study, the extracorporeal circuit volume represented approximately 23–31% of the estimated circulating blood volume of the included cats. Even though the circuit was primed with whole blood, this relatively large extracorporeal volume may have increased susceptibility to hematologic instability during renal replacement therapy. The consistent decline in hematocrit observed in all three cats in this series aligns with previous reports describing anemia in small animal continuous renal replacement therapy patients [4].

Despite repeated whole blood transfusions in all cases, hematocrit declined progressively during the CRRT period. This observation supports the concept that CRRT-associated anemia cannot be fully explained by inadequate transfusion support or erythropoietin deficiency alone [8,16]. This interpretation is further supported by the absence of filter clotting or clinically apparent extracorporeal circuit blood loss during treatment. Therefore, overt circuit-related blood loss alone is unlikely to explain the progressive decline in hematocrit observed in these cats, suggesting that multiple concurrent mechanisms contributed to anemia development.

Renal parameters demonstrated marked improvement immediately following CRRT but showed variable rebound patterns among individual cats. In two cases, intermittent increases in blood urea nitrogen and creatinine following initial improvement were consistent with dialysis-dependent acute kidney injury, characterized by ongoing uremic generation in the setting of intermittent extracorporeal clearance [19,20]. These fluctuations emphasize that hematologic trends observed during CRRT occur within a highly unstable metabolic and inflammatory environment rather than a steady physiologic state.

Notably, increases in absolute reticulocyte counts were observed in two cats (Cat 1 and Cat 2) after completion of CRRT, indicating that erythropoietic activity was not completely suppressed during treatment and may recover as inhibitory factors diminish. Differences in reticulocyte response among cases likely reflect interindividual variability in disease severity, inflammatory and infectious burden, and concurrent therapies. In particular, the comparatively modest reticulocyte response observed in Cat 3, which had concurrent inflammatory and infectious disease among other confounders, may reflect inflammation-mediated suppression of erythropoiesis [21,22]. All three cats received darbepoetin alfa and molidustat in combination with CRRT and transfusion support; given this overlap of interventions and the short observation period, the individual contribution of any single treatment to the observed reticulocyte responses cannot be determined.

At the time of treatment, molidustat was used for the management of nonregenerative anemia associated with CKD in cats [23], but not specifically for use in ACKD or during CRRT; therefore, its use in all three cases was off-label. The decision to administer molidustat was based on the presence of underlying CKD-associated anemia and the clinician’s judgement that potential benefits in supporting erythropoiesis outweighed the risks in these critically ill cats.

Although anemia progressed during active CRRT, no abrupt worsening or clinically significant adverse events temporally associated with molidustat administration were observed. Following discontinuation of CRRT, stabilization of hematocrit and evidence of erythropoietic activity were noted in some cats. However, this study was not designed to evaluate the efficacy of molidustat, and no conclusions regarding its contribution to hematologic recovery can be drawn. Observed reticulocyte responses cannot be clearly separated from physiologic recovery following CRRT or from the effects of transfusion and ESA use.

Interpretation of the hematologic trajectory in Cat 3 is particularly limited by substantial clinical confounding. In addition to severe AKI superimposed on pre-existing CKD, this cat had multiple concurrent systemic and infectious/inflammatory conditions and underwent repeated CRRT and three blood transfusions while receiving darbepoetin alfa and molidustat. Consequently, the observed hematologic changes cannot be attributed specifically to molidustat. During prolonged hospitalization, this cat also developed respiratory and ocular signs with PCR-confirmed feline herpesvirus, feline calicivirus, and *Mycoplasma* spp. infection; these findings may have contributed to the inflammatory and hematologic milieu, but their independent contribution to the anemia observed earlier in the treatment course cannot be quantified from the available retrospective data.

Overall, these findings provide descriptive insight into the hematologic course of cats undergoing CRRT and highlight the predictable progression of anemia despite transfusion support. The observations underscore the need for future prospective studies to better define anemia pathophysiology and to systematically evaluate anemia management strategies in feline patients receiving CRRT. Clinicians should anticipate progressive anemia during feline CRRT and monitor hematologic parameters proactively, even in the presence of transfusion support.

### Limitations

This study has several important limitations. First, the small number of included cats and the retrospective observational design preclude statistical analysis and causal inference. Findings are descriptive in nature and should not be generalized beyond similar clinical contexts. This limitation reflects the inherent challenges of studying critically ill small animal populations undergoing CRRT [19,20].

Second, the absence of a control group prevents differentiation of the individual effects of CRRT, whole blood transfusion, darbepoetin alfa, and adjunctive molidustat administration, all of which were administered concurrently or in close temporal proximity in each cat. Consequently, it cannot be determined whether observed hematologic changes were attributable to any specific intervention or to post-CRRT physiologic recovery. The three cats also differed in the number of CRRT sessions and cumulative extracorporeal exposure (one session in Cat 1 versus three sessions each in Cats 2 and 3), and laboratory sampling was performed according to clinical need rather than a standardized prospective schedule, further limiting direct comparison among cases. Similar limitations have been reported in both veterinary and human CRRT studies [7,19].

Donor packed cell volume (PCV) was not retained in the reviewed records for Cat 1 or Cat 2. For Cat 3, a donor PCV of 33% (blood-bank-supplied blood) was documented on a transfusion monitoring sheet from a later transfusion (26 October) administered during this cat’s prolonged hospitalization, outside the primary CRRT-associated treatment period described in Results. In clinical practice at our institution, feline whole blood transfusions are volume-limited (typically up to approximately 40 mL per session, reflecting the maximum volume that can safely be collected from a donor cat) and are administered proactively, in anticipation of CRRT-associated anemia, rather than reactively in response to a specific pre-transfusion PCV threshold or evidence of hemolysis. Accordingly, the post-transfusion HCT changes reported here should not be interpreted as evidence of transfusion reaction or donor-related hemolysis, and the continued decline in HCT despite transfusion is considered attributable to the severity and volume demands of CRRT itself rather than to transfusion inadequacy.

Third, variables that may influence anemia development and erythropoietic response, including anticoagulation protocols, blood sampling frequency, inflammatory status, and iron metabolism indices, were not systematically assessed due to limitations of retrospective medical records. The absence of these data limits mechanistic interpretation of the observed trends [24]. Furthermore, mechanistic biomarkers, including serum iron concentration, ferritin, transferrin saturation, endogenous erythropoietin concentration, hepcidin, and inflammatory cytokines, were not available [8,9]. Consequently, the mechanisms proposed in this study should be regarded as hypothesis-generating rather than definitive.

Finally, the cats included represented a heterogeneous population of critically ill patients with different apparent AKI etiologies (undetermined in Cat 1, postrenal obstruction in Cat 2, and multifactorial in Cat 3, including concurrent hepatobiliary, pancreatic, and later respiratory/infectious disease), varying comorbidities, and differing degrees of renal dysfunction. Accordingly, results should be interpreted within the context of similarly severe clinical presentations rather than extrapolated to all feline patients receiving CRRT. Nevertheless, serial hematologic observations in feline patients undergoing CRRT remain exceedingly scarce, and these findings provide preliminary data that may inform the design of future prospective studies.

## 5. Conclusions

This retrospective observational study describes temporal hematologic and renal trends in three critically ill cats undergoing continuous renal replacement therapy for severe acute kidney injury with evidence of pre-existing chronic kidney disease, during which molidustat was used as part of multimodal anemia management. Despite repeated whole blood transfusions, progressive anemia occurred consistently during the CRRT period, while evidence of erythropoietic recovery was observed in two cats following discontinuation of CRRT. Because of the concurrent use of whole blood transfusion, darbepoetin alfa, and CRRT itself, together with critical illness, heterogeneous comorbidities, and a short observation period, the independent hematologic effect of molidustat cannot be determined from these observations.

These findings emphasize the complex and multifactorial nature of anemia in feline patients receiving CRRT and suggest that transfusion support alone may be insufficient to prevent anemia progression in this high-risk clinical setting. The descriptive data presented herein are hypothesis-generating rather than confirmatory, provide clinically relevant insight into anemia progression and management challenges during feline CRRT, and may inform the design of future prospective, controlled studies. To our knowledge, this study represents one of the first reports describing serial hematologic trajectories during CRRT in cats and provides preliminary observational data to support future investigations into CRRT-associated anemia in feline patients.

## Figures and Tables

**Table 1:** Baseline characteristics and treatment overview of three cats undergoing CRRT and receiving molidustat.

Variable	Cat 1	Cat 2	Cat 3
Breed	Domestic shorthair	Persian	Domestic shorthair
Age (years)	8	12	10
Sex	Spayed female	Neutered male	Neutered male
Body weight (kg)	3.7	3.78	3.5
Primary diagnosis	ACKD	ACKD with left ureterolithiasis (partial unilateral obstruction) and bilateral renal microlithiasis/mineralization	ACKD
Reason for CRRT	Severe azotemia, uremia	Severe azotemia, uremia	Severe azotemia, uremia
Number of CRRT sessions (*n*)	1	3	3
Whole blood transfusion (type A)	1 × 40 mL (Day 0)	3 × 40 mL (Days 0, 2, 5)	40 + 40 + 50 mL (Days 0, 2, 6)
ESA administration	Darbepoetin alfa (1 µg/kg SC, single dose)	Darbepoetin alfa (1 µg/kg SC, single dose)	Darbepoetin alfa (1 µg/kg SC, Day 0)
Molidustat administration	Yes	Yes	Yes
Start of molidustat (relative to CRRT)	Post-CRRT (Day 2)	At initiation of CRRT 1 (Day 0)	At initiation of CRRT 1 (Day 0)

ACKD, acute-on-chronic kidney disease; CRRT, continuous renal replacement therapy; ESA, erythropoiesis-stimulating agent. Body weight for Cat 2 is the value recorded at admission; a subsequent physical examination on the day of the first CRRT session (14 March) recorded 4.01 kg. Day numbering is relative to Day 0, defined as the day of the first CRRT session for each cat. All transfusions used type A whole blood; Cat 1 received blood from a live donor cat, and Cats 2 and 3 received blood-bank-supplied blood.

**Table 2:** Summary of hematologic and renal parameters in three cats undergoing CRRT and receiving molidustat.

Variable	Cat 1	Cat 2	Cat 3
Day 0 (pre-CRRT) HCT (%)	38.6	31.7	39.9
Day 0 (pre-CRRT) reticulocyte count (K/µL)	8.1	5.5	102.2
Day 0 (pre-CRRT) BUN (mg/dL)	210	138.5	>140 ^a^
Day 0 (pre-CRRT) creatinine (mg/dL)	19.0 ^b^	7.4	6.97
Day 0 (pre-CRRT) phosphorus (mg/dL)	21.8	11.9	7.31
Lowest HCT (%), date	19 (24 February)	21.9	20.8 (18 October)
Peak reticulocyte count (K/µL), date	61.3 (23 February)	59.1	44.7 (18 October) ^c^
Peak BUN (mg/dL)	210	160	>140 ^a^
Peak creatinine (mg/dL)	19.0	8.09 ^d^	6.97
Peak phosphorus (mg/dL)	21.8	11.9	7.31
Post-CRRT renal trend	Sustained improvement	Rebound pattern	Gradual stabilization
Overall anemia pattern	Progressive, regenerative	Progressive, regenerative	Progressive, mildly regenerative

^a^ BUN exceeded the analyzer’s upper measurement limit (>140 mg/dL) on multiple occasions during the hospitalization of Case 3; the true value may therefore have been higher than the highest quantifiable reading. ^b^ Admission creatinine for Cat 1 was confirmed directly against the clinical record by the authors; other proximate measurements in the record were not substituted for this admission value. ^c^ A single reticulocyte count of 131.4 K/µL, recorded 6 h into the first CRRT session in Case 3, was excluded as a presumed analyzer artifact; this value was inconsistent with the measurements immediately before (13.2 K/µL) and after (9.9 K/µL) it and coincided with marked leukocytosis, a recognized source of interference in automated reticulocyte counting. ^d^ Peak creatinine for Cat 2 was recorded on 19 March (Day 5), reflecting rebound azotemia following the second CRRT session, rather than at Day 0. The Lowest HCT values in this table were obtained from serial point-of-care (GEM5000 blood gas analyzer) measurements and may not correspond to the same sampling method as the Day 0 CBC-based HCT values listed above; see Section 2.4 for details on data collection.

## Data Availability

The data supporting the findings of this study are available from the corresponding author upon reasonable request. The data are not publicly available due to privacy and confidentiality considerations associated with clinical records of client-owned animals.

## Supplementary Materials

The following supporting information can be downloaded at: https://www.mdpi.com/article/10.3390/vetsci13090973/s1, Detailed individual clinical data, including CRRT indications, treatment timelines, transfusion history, erythropoietic support, and serial laboratory values, are available as Supplementary Material: Table S1: Signalment, primary diagnoses, and indications for continuous renal replacement therapy (CRRT) in three cats; Table S2: Hematologic parameters, transfusion history, and erythropoietic support in three cats undergoing CRRT with concurrent molidustat administration; Table S3: Serial renal biochemical parameters demonstrating response to CRRT and post-dialysis rebound in three cats.
