# Supplementary material for: Hematologic and Renal Trends in Cats Undergoing Continuous Renal Replacement Therapy: A Retrospective Case Series with Molidustat Use
Source: Vet Sci. 2026 Sep 16;13(9):973. doi: 10.3390/vetsci13090973 (PMC13612000; doi:10.3390/vetsci13090973)
Supplement: Supplementary file 1 [file vetsci-13-00973-s001.zip › vetsci-4522504-supplementary_corrected.pdf]

## Supplementary Data

**Supplementary Table S1.** Signalment, primary diagnoses, and indications for continuous renal replacement therapy (CRRT) in three cats.

| Case | Signalment | Key presenting signs | Primary diagnoses | Indication for CRRT | CRRT sessions (n) | Outcome |
|---|---|---|---|---|---|---|
| Case 1 | 8y FS, 3.7 kg, DSH | Anorexia, persistent vomiting, severe dehydration | ACKD, severe azotemia, uremic gastritis, anemia | Rapid worsening azotemia refractory to medical therapy; inability to tolerate oral intake | 1 | Post-CRRT diuresis; Cr improved to 2.66 mg/dL at discharge |
| Case 2 | 12y NM, 3.78 kg, Persian | Lethargy, obtunded mentation | ACKD, left ureteral calculi with partial unilateral obstruction, bilateral renal microlithiasis, HCM stage C, anemia | Severe azotemia with postrenal component; limited fluid therapy due to cardiac disease; metabolic stabilization prior to intervention | 3 | Post-CRRT stabilization with recurrent rebound; dialysis-dependent AKI |
| Case 3 | 10y NM, 3.5 kg, DSH | Stuporous mentation, systemic deterioration | ACKD, uremic encephalopathy, hyperammonemia, multifactorial AKI with concurrent hepatobiliary/pancreatic disease, small-volume abdominal free fluid | Life-threatening uremia with neurologic compromise and hyperammonemia | 3 | Partial neurologic improvement; poor overall prognosis |

**Supplementary Table S2.** Hematologic parameters, transfusion history, and erythropoietic support in three cats undergoing CRRT with concurrent molidustat administration.

| Case | Whole blood transfusions | ESA use | Molidustat dose & timing | Lowest HCT (%) | Peak reticulocyte count (K/µL) | Hematologic interpretation |
|---|---|---|---|---|---|---|
| Case 1 | 1 × 40 mL (type A) | DPO 1 µg/kg once (Day 2) | 5 mg/kg SID PO, started Day 2 (post-CRRT) | 19 | 61.3 | Progressive but regenerative anemia |
| Case 2 | 3 × 40 mL (type A) | DPO 1 µg/kg once | 5 mg/kg SID PO, started Day 0 (at CRRT | 21.9 | 59.1 | Persistent regenerative |

| | | | | | | |
|---|---|---|---|---|---|---|
| | | (Day 1) | initiation) | | | anemia despite CRRT |
| Case 3 | 40 + 40 + 50 mL (type A) | DPO 1 µg/kg once (Day 0) | 5 mg/kg SID PO, started Day 0 (at CRRT initiation) | 20.8 | 44.7 | Mildly regenerative anemia, potentially influenced by concurrent inflammatory/infectious disease |

**Supplementary Table S3.** Serial renal biochemical parameters demonstrating response to CRRT and post-dialysis rebound in three cats.

| **Case** | **Peak BUN (mg/dL)** | **Peak creatinine (mg/dL)** | **Post-CRRT pattern** | **Interpretation** |
|---|---|---|---|---|
| Case 1 | 210 | 19.0 | Rapid decrease with transient rebound | Severe AKI with partial recovery |
| Case 2 | 160 | 8.09 | Repeated decrease and rebound | Dialysis-dependent AKI |
| Case 3 | >140 | 6.97 | Marked fluctuations | Severe AKI with systemic complications |

Abbreviations: ACKD, acute-on-chronic kidney disease; CRRT, continuous renal replacement therapy; DPO, darbepoetin alfa; ESA, erythropoiesis-stimulating agent; HCT, hematocrit.
